# Biotechnological Approaches for Biomass and Lipid Production Using Microalgae *Chlorella* and Its Future Perspectives

**DOI:** 10.4014/jmb.2209.09012

**Published:** 2022-10-21

**Authors:** Sujeong Je, Yasuyo Yamaoka

**Affiliations:** Division of Biotechnology, The Catholic University of Korea, Bucheon 14662, Republic of Korea

**Keywords:** *Chlorella*, biotechnology, lipids, microalgae, biomass, phycoremediation

## Abstract

Heavy reliance on fossil fuels has been associated with increased climate disasters. As an alternative, microalgae have been proposed as an effective agent for biomass production. Several advantages of microalgae include faster growth, usage of non-arable land, recovery of nutrients from wastewater, efficient CO_2_ capture, and high amount of biomolecules that are valuable for humans. Microalgae *Chlorella* spp. are a large group of eukaryotic, photosynthetic, unicellular microorganisms with high adaptability to environmental variations. Over the past decades, *Chlorella* has been used for the large-scale production of biomass. In addition, *Chlorella* has been actively used in various food industries for improving human health because of its antioxidant, antidiabetic, and immunomodulatory functions. However, the major restrictions in microalgal biofuel technology are the cost-consuming cultivation, processing, and lipid extraction processes. Therefore, various trials have been performed to enhance the biomass productivity and the lipid contents of *Chlorella* cells. This study provides a comprehensive review of lipid enhancement strategies mainly published in the last five years and aimed at regulating carbon sources, nutrients, stresses, and expression of exogenous genes to improve biomass production and lipid synthesis.

## Introduction

Microalgae have recently drawn considerable attention for their high potential to produce valuable compounds as well as their applications in biodiesel production, phycoremediation, and dietary supplements. As a source of bioenergy raw materials that can be used to produce biofuels, microalgae are a unique bioresource that has been proposed as a solution to combat energy shortages and alleviate problems associated with global warming [1, 2]. Compared to terrestrial plants, microalgae have tremendous potential as a bioresource with greater biomass productivity [3, 4]. Typically, 10-20% of the biomass derived from microalgae consists of fatty acids that can be used as raw materials for bioenergy [5]. However, there are some limitations to the industrial applications of microalgae bioenergy [6]. The biomass produced through microalgae cultivation is harvested using processes such as centrifugation and filtration [7, 8]. Significant losses and production costs are incurred during harvest [7, 8]. Therefore, solutions to reduce the losses and production costs associated with harvesting processes are essential [9].

*Chlorella* belongs to the Chlorophyta division and consists of small, non-motile, spherical unicellular microalgae with a single chloroplast [10]. *Chlorella* mainly lives in freshwater, but a few species are also found in the marine environment. They are autospores and mainly reproduce asexually by mitosis [11]. *Chlorella* species have been used as a bioresource because of their valuable molecules. Although they were initially considered a food resource owing to their high protein content [12], they have more recently been used for biofuel production [13]. *Chlorella* species have high adaptability to environmental variations [14], and these characteristics make *Chlorella* the most effective microalgae for the generation of bioresources. In addition, *Chlorella* species can survive and accumulate products using wastewater, making them an exciting target of study in the phytoremediation field [15, 16].

For example, lipids accumulated by microalgae can be used as feedstock for biodiesel production, and microalgal oils can be used in the food industry [17, 18]. Many studies have shown the importance of cultivation conditions for microalgal growth and lipid accumulation. Nutrients [19, 20], high salinity [21, 22], metal ions [23], light intensity, temperature, pH, and abiotic/biotic treatments are regarded as critical parameters for microalgal growth and lipid accumulation. This review presents updated research on *Chlorella* biomass and lipid production, published mainly in the last five years, and discusses the subsequent progress and perspectives.

## Nutrients

### Carbon Source for Cultivation of *Chlorella*

Photoautotrophic growth of microalgae requires inorganic carbon as a carbon source for growth, which relies on light as a sole energy source. The application of organic carbon sources can be divided into two types depending on light's presence (mixotrophic) or absence (heterotrophic). *Chlorella* strains can grow under photoautotrophic, heterotrophic, and mixotrophic conditions, allowing them to shift in response to changes in the environment. Many studies have been performed to reveal the cultivation method with high efficiency under either photoautotrophic, mixotrophic, or heterotrophic conditions [24-27].

## Photoautotrophic Mode

**Fig. 1:**
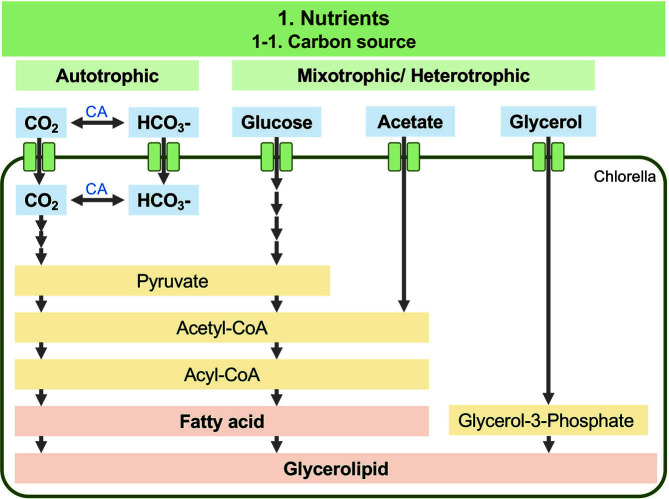
Overview of the putative metabolic pathways for lipid biosynthesis under heterotrophic cultivation in *Chlorella*. Uptake of glucose, acetate, and glycerol to produce lipids. Intermediate products and other metabolic biosynthesis pathways were omitted from this metabolic pathway.

In photoautotrophic cultivations, the only source of carbon for photosynthesis comes from the available atmospheric CO_2_ (Fig. 1). The photobioreactor system is capable of photoautotrophic cultivation of *Chlorella*, and several studies investigated the ability for lipid production with the combination of photoautotrophic cultivation and nitrogen depletion, which will be discussed later. Amaral *et al*. optimized photoautotrophic cultivation of *Chlorella* in a photobioreactor with statistical analysis, and increased the biomass and lipid productivity of *C. minutissima* at least 1.42-fold and 2.43-fold (5.72 mg/l/day), respectively, in a medium with reduced nutrient availability [28]. Singh *et al*. developed a two-stage photoautotrophic lipid production strategy in a sintered disk photobioreactor [29]. Initially, *C. pyrenoidosa* was incubated with sufficient nutrients and further treated with nitrogen starvation for lipid induction, resulting in 410 mg/l/day lipid productivity [29]. This lipid productivity is higher than those obtained from different photoautotrophic cultivation strategies of different algal strains (Table 1 in [29]). Investigating proper light intensity and CO_2_ levels is also essential in photoautotrophic cultivation. *C. sorokiniana* AM-02 was cultured under high photosynthetic photon flux density (PPFD) conditions and CO_2_ gas levels. The preferred high PPFD and optimal CO_2_ levels were 1,000-1,400 μmol photons/m^2^/s and 0.5-2.0% (v/v), respectively [30]. According to our research, not so many reports intend to improve the photoautotrophic condition for *Chlorella* cultivation within these five years due to low biomass and lipid productivity compared to heterotrophic or mixotrophic conditions.

## Heterotrophic Mode

Although microalgae can utilize inorganic carbon sources for photosynthesis, the biomass productivity of microalgae is low and limited [31]. Biomass productivity can be improved under heterotrophic conditions compared to the basal photoautotrophic culture conditions. Morowvat *et al*. optimized the growth conditions of naturally isolated *C. vulgaris* strain in BG-11 medium in a flask and bioreactor. The total biomass and lipid content in heterotrophic culture with glucose were improved 3.5-fold and 9.3-fold, respectively, compared to the basal photoautotrophic culture condition in the shake flask experiment [32]. In the bioreactor experiment, total biomass and lipid concentration or density also increased to 4.95 and 2.18 g/l, respectively, during five days of the experiment compared to its basic photoautotrophic culture [32]. Kim *et al*. developed heterotrophic cultivation conditions using statistical assessment to explore the full potential of *Chlorella* sp. HS2, which was isolated for the ability to achieve extraordinary culture density (5.91 g/l) and biomass productivity (656.7 mg/l/day) under photoautotrophic conditions [33]. The cultivation with a 5-L fermenter under heterotrophic conditions using glucose resulted in significantly improved biomass productivity (5.37 g/l/day), and total lipid productivity (0.86 g/l/day) was achieved [33]. Their trial was vastly superior to the performance in most previous works involving the heterotrophic fermentation of green algae (Table 3 in [33]). Thus, heterotrophic cultivation allows us to produce much higher biomass and lipid yields than photoautotrophic mode. However, specialists have not concluded which option, photoautotrophic or heterotrophic, is the most economical. Recent reports discussed possible future scenarios in which the cost of heterotrophic production of microalgae on an industrial scale would be comparable to autotrophic production [34].

## Mixotrophic Mode

In the mixotrophic mode, photoautotrophic metabolism is integrated with heterotrophic metabolism. Recent studies successfully increased the *Chlorella* biomass and lipid productivity by using various organic carbon sources for cultivation, such as glucose, acetate, or glycerol (Fig. 1). Of these, many previous publications concluded that glucose is an efficient trigger to increase biomass productivity of the microalgae [35-37]. Recently, Yun *et al*. evaluated the applicability and usability of 10 g/l glucose as an organic carbon source for *C. vulgaris* and *C. sorokiniana* under heterotrophic and mixotrophic conditions [38]. As a result of optimization of culture conditions, mixotrophic conditions provided the highest lipid content (68.80%) in *C. vulgaris* KNUA104 and the highest biomass production (4.73 mg/l/day) in *C. sorokiniana* [38]. Ward and Rehmann optimized various nutrients for mixotrophic cultivation, including glucose, sodium nitrate, and magnesium sulfate, by the response surface methodologies, which can evaluate complex relationships, resulting in overall lipid productivity of 383 mg/l/day with 18.8 g/l glucose as a carbon source [39]. Thus, glucose seems to be a promising candidate as a carbon source for the mixotrophic cultivation of algal cells.

Acetic acid is preferentially adsorbed by the microalgal cells and directly converted into acetyl-CoA, achieving higher efficiency of lipid production. León-Vaz *et al*. used 100 mM (6 g/l) acetic acid from the oxidized wine waste lees for mixotrophic cultivation of *C. sorokiniana*, and lipid productivity was 193.37 mg/l/day [40]. Liu *et al*. used 10 g/l sodium acetate as organic carbon for mixotrophic cultivation of *C. pyrenoidosa* to obtain the maximum production of total lipid [41].

*Chlorella* cultivation in a mixotrophic mode with glycerol enhanced the overall biomass concentration and lipid accumulation [42-44]. Recently, Rana and Prajapati showed that supplementation of glycerol (3 g/l) in synthetic wastewater (SWW) could enhance lipid accumulation (30.76% dry weight basis) in *C. pyrenoidosa* compared to control (without glycerol, 13.16% dry weight basis) [45].

Chai *et al*. compared the effect of four monosaccharides (glucose, fructose, galactose, and xylose) on *Chlorella* growth. *Chlorella* medium with fructose promoted *C. sorokiniana* growth to a much lesser extent than glucose, whereas supplementation with galactose had no effect, and supplementation with xylose inhibited growth [46].

The question is which carbon source will provide the best lipid productivity. Glucose is first catabolized into glucose-6-phosphate and converted to pyruvate through an anaerobic glycolysis process. Furthermore, it is converted into acetyl-CoA, which is subsequently utilized in the TCA cycle for energy production or as a precursor for fatty acid synthesis (Fig. 1); therefore, both biomass and lipid production can be accelerated. On the other hand, acetate is a simple substrate necessitating only one or two activation steps at the expense of one ATP molecule to produce acetyl-CoA [47]. Perez-Garcia *et al*. evaluated eleven known carbon sources for the cultivation of *C. vulgaris* (Beij.) and found that the best growth rate was provided by acetate cultivation and the second by glucose cultivation [35, 48]. In principle, the uptake of glucose and ammonium during the mixotrophic growth would decrease pH [49, 50], while acetate consumption and photosynthesis increase pH [51, 52]. To solve this problem, Xie *et al*. proposed Glucose-Acetate-Phosphorus (GAP) medium, which can maintain pH during cultivation [53], and might be a promising alternative for mixotrophic cultivation.

## Heterotrophic Mode vs. Mixotrophic Mode

Some researchers compared the effects of conditions in heterotrophic and mixotrophic cultivation modes for *Chlorella* biomass or lipid productivity. Li *et al*. reported that heterotrophic cultivation produced the maximum biomass productivity of 134.9 mg/l/day and maximum lipid productivity (42.4 mg/l/day), which were much higher than those under mixotrophic condition [54]. Canelli *et al*. found that the heterotrophic mode maximized lipid productivity (32.7% DCW), and the mixotrophic condition induced a nutritionally favorable fatty acid profile and higher concentrations of carotenoids and phenolics with less lipid quantity (24.2% DCW) [55]. Applying light to heterotrophic cultivation (mixotrophy) might induce oxidative stress, increasing the carotenoid content [56]. On the other hand, Yun *et al*. reported that *Chlorella* biomass productivity and lipid yield under mixotrophic conditions (3.02 g/l/day and 1.86 g/l, respectively) were higher than the heterotrophic conditions (1.78 g/l/day, 0.54 g/l, respectively) [38].

It remains a question as to which trophic mode is best for growing *Chlorella* on a global industrial scale. The *Chlorella* market is mainly segmented into autotrophic and heterotrophic, and the heterotrophic segment is expected to grow at the highest compound annual growth rate (CAGR) during the forecast period [57]. It is mainly used due to its higher cell concentration, higher productivity, low risk of contamination, lower water consumption, low space usage, and purity of biomass.

## Nitrogen Source for Cultivation of *Chlorella*

**Fig. 2:**
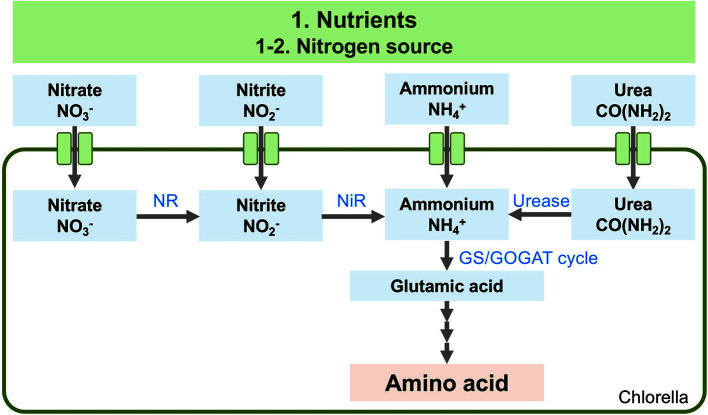
Overview of the putative metabolic pathways for nitrogen assimilation in *Chlorella*. Uptake of nitrate, nitrite, ammonium, and urea to produce amino acids. Intermediate products and other metabolic biosynthesis pathways were omitted from this metabolic pathway. NR, nitrate reductase: NiR, nitrite reductase GS, glutamine synthetase; GOGAT; glutamine oxoglutarate aminotransferase.

Nitrogen is one of the essential nutrients for microalgal cultivation. Nitrogen can be delivered in various forms to the culture, such as nitrate NO_3_^-^, nitrite NO_2_^-^, ammonium NH_4_^+^, and urea CO(NH_2_)_2_. Although ammonium (NH_4_^+^) can be directly assimilated into amino acids via the GS/GOGAT cycle [58, 59], nitrate (NO_3_^-^) needs to be reduced to nitrite (NO_2_^-^) in the cytosol, after which it is immediately reduced to ammonium in chloroplasts or plastids [60] (Fig. 2). Thus, ammonium is more efficient than nitrate as a nitrogen source. However, ammonium can be toxic to many organisms, particularly plants and oxygenic photosynthetic microorganisms [61, 62]. Here, we mentioned recent trials to investigate the nitrogen sources that allow better cultivation and growth of *Chlorella*.

## Nitrate vs. Nitrite

Pozzobon *et al*. cultivated *C. vulgaris* using mixed nitrate and nitrite as a nitrogen source to optimize the ratio of nitrate to nitrite. Nitrite addition triggered a growth rate inhibition, and nitrite uptake remained constant at a low level [63]. On the other hand, nitrate uptake rate was correlated with nitrate content in the culture medium [63]. Mutlu *et al*. previously showed that cultivations with nitrate produced higher protein content (51%) compared to those with nitrite (41%) [64]. Recently, different *Chlorella* strains also reported better growth with nitrate supplementation [65].

A few studies showed better lipid productivity using nitrite as a nitrogen source. Zhan *et al*. previously reported that nitrite-nitrogen (NO_2_-N) was the best among the nitrogen sources for the HQ growth and lipid accumulation potential of *Chlorella* sp. Nitrate-nitrogen (NO_3_-N) and urea-nitrogen (Urea-N) also contributed to algal growth and lipid accumulation potential at a lesser level, but ammonium nitrogen (NH_4_-N) and N-deficiency instead caused inhibitory effects in this *Chlorella* strain [66]. Li *et al*. also reported that 200 μmog/l nitrite provided 3.0 mg/l/day lipid productivity in *Chlorella* sp. L38. By contrast, the average lipid productivity in the medium containing 200 μmog/l nitrite + nitrate resulted in 1.15 mg/l/day [65]. Such increases in lipid content in nitrite medium in these studies might be due to the induction of nitrogen deficiency condition, which is beneficial for lipid accumulation [67], since nitrite seems to be barely taken up by most *Chlorella* cells [63].

## Urea

The consumption of either nitrate or ammonium by microalgae causes a change in medium pH as they grow. Davis *et al*. reported that glycine and urea were organic nitrogen sources without a drastic increase in pH fluctuations in the medium [68]. Several reports showed that *Chlorella* species could grow faster in urea than in nitrate and ammonium as nitrogen sources. When urea was the nitrogen source, the highest dry cell weight (2.86 g/l), biomass productivity (345 mg/l), and specific growth rate (1.903/day) were obtained in *Chlorella* sp. GN1 culture [69]. Additionally, Nayak *et al*. reported that urea best promoted the biomass production, specific growth rate, and biomass productivity of *Chlorella* sp. HS2 among all nitrogen sources [70].

## Ammonium

Ammonia nitrogen includes the ionized (ammonium, NH_4_^+^) and unionized form (ammonia, NH_3_, toxic to aquatic organisms). Unlike nitrate NO_3_^-^, when ammonium NH_4_^+^ is utilized, microalgae spend less energy on its assimilation, and ammonium is directly incorporated into amino acids [71]. However, excessive amounts of ammonium are toxic to algae due to the damaging effects on photosynthesis [61, 72]. This is because ammonium directly induces photodamage to PSII rather than affecting the repair of photodamaged PSII [73, 74].

*Chlorella* can use ammonium for growth, making it possible to use this alga for bioremediation to remove ammonium [75]. Ziganshina *et al*. reported that the highest growth rate (1.26/day) was observed in modified Bold’s basal medium (BBM) with ammonium, while the growth rate in BBM with nitrate was only 1.07/day [76]. Thus, although *Chlorella* strains seem tolerant to ammonium, the degree of growth inhibition by ammonium varies between the strains [71, 77, 78]. Wang *et al*. compared the tolerance of green algae to ammonium using ten *Chlorella* strains. As a result, FACHB-1563 had the highest tolerance to ammonium among all strains tested, suggesting that FACHB-1563 might be able to remove excess ammonium from wastewater for bioremediation [74]. Thus, different nitrogen sources have different effects on the physiological indexes of *Chlorella* strains.

In photosynthetic eukaryotes, nitrogen assimilation is performed by nitrate or nitrite transports. From the structural point of view, three families of proteins are involved in nitrate or nitrite transport in microalgae: *NRT1* (nitrate NO_3_^-^ transporter), *NRT2* (nitrate NO_3_^-^ nitrite NO_2_^-^ transporter), and NAR1 (nitrite NO_2_^-^ transporter) [58]. Interestingly, although *Chlorella* sp. NC64A has a complete set of all the genes needed for nitrate assimilation, the strain can use ammonium or amino acids but not nitrate or nitrite [79]. Additional complexity is the genetic variation of nitrogen assimilation genes among *Chlorella* strains. *Chlorella* NC64A contains two *NRT1* and two *NRT2* genes, but *C. paradoxa* conserves two *NRT2* genes (Table 1 in [58]). Hence, we must carefully select nitrogen sources depending on the *Chlorella* strains used and the purpose of cultivation based on genetic information.

## Phycoremediation: Wastewater as a Nutrient Source for Cultivation of *Chlorella*

Phycoremediation refers to remediation with the help of algae. Using wastewater to grow microalgae as a nutrient source would decrease the cultivation costs and purify polluted water. Food waste also represents a valuable carbon source for algal cultivation and can improve the production of microalgal biomass and valuable oleochemicals. Arora and Philippidis utilized 25% sweet sorghum bagasse (SSB) hydrolysate and achieved the highest biomass and lipid productivity (3.44 g/l and 120 mg/l/day, respectively) under mixotrophic conditions compared to heterotrophic and photoautotrophic conditions [80]. Wang *et al*. used 10 g/l glucose from food waste hydrolysis for mixotrophic cultivation of *Chlorella* sp. GY-H4, resulting in a 6.1 g/l biomass yield with a 2.5 g/l lipid yield [81]. The cultivation of *C. sorokiniana* in palm oil mill effluent (POME) using a novel designed photobioreactor has brought enhancement in biomass production (409 mg/l/day), excellent lipid content (14.43%, DCW), as well as effective POME remediation [82]. Chen *et al*. investigated the effects of sugarcane bagasse hydrolysate (SCBH) carbon sources on cell growth and fatty acid accumulation in *Chlorella protothecoides*. With the medium containing SCBH (20 g/l sugar concentration), the highest biomass and fatty acid yield were 10.7 g/l and 0.55 g/l, respectively, which was significantly higher than that in the culture using glucose [83]. Taken together, utilizing these food waste hydrolysates seems to create potential industrial applications for sustainable *Chlorella* biomass and lipid production.

Using forest residues for biofuel production has attracted interest due to the generation of additional revenue and reduction of greenhouse gas emissions. Vyas *et al*. utilized cellulose-rich pretreated solids from spruce biomass to grow and produce lipids in oleaginous microalgae. They cultivated microalgae in a medium containing (20 g/l) glucose obtained from spruce hydrolysate, which resulted in the production of biomass (8.28 g/l at C/N 60) and lipid synthesis (3.61 g/l at C/N 60) after 72 h of cultivation [84].

The commercial seafood processing industry generates large quantities of solids and wastewater. Seafood processing wastewater (SPW) usually contains high concentrations of nutrients, indicating that SPW could be an alternative nutrient source for microalgae cultivation. Jain *et al*. cultivated *C. vulgaris* in SPW under mixotrophic conditions [85]. The biomass productivity and lipid content accounted for 264.58 mg/l/day and 38% (DCW), respectively, at a 10% CO_2_ supply [85]. Gao *et al*. treated *Chlorella* sp. with aerated seafood processing wastewater, and higher biomass productivity (77.7 mg/l/day) and higher lipid productivity (20.4 mg/l/day) were obtained compared to those in SPW [86], indicating that the aeration pretreatment is essential to reduce the amount of toxic unionized ammonia in SPW.

Sewage wastewater treatment with microalgae cultivation is an eco-friendly process. Saranya and Shanthakumar evaluated the remediation of combined sewage and tannery effluent under different dilutions. The maximum biomass yield was achieved at 20% tannery effluent and 80% sewage effluent (20% tannery effluent diluted with sewage), resulting in 3.25 g/l and 2.84 g/l in *C. vulgaris* and *Pseudochlorella pringsheimii*, respectively. Between the two species, *P. pringsheimii* showed high lipid accumulation potential of 25.4% (dry weight basis) compared to *C. vulgaris* (9.3%) at 20% tannery effluent diluted with sewage (Fig. 3 in [87]). Azam *et al*. investigated the production of *Chlorella* biomass and nutrient removal efficiencies with a 50% concentration of open sewage contaminated channel wastewater (OSCCW), which contributed to the biomass (60.1 and 56.5 g/l) and lipid content (20.8 and 17.5 mg/l/day) in *C. vulgaris* and *C. pyrenoidosa*, respectively [88]. Anaerobic hydrolysis and acidification of complex organic wastes are common wastewater treatment methods. There are three widely recognized fermentation types in a mixed culture of acidogenesis: butyric acid type, propionic acid type, and ethanol type. Of these, the highest lipid content (25.4% DCW) of *Chlorella* sp. UJ-3 was achieved in the butyrate-type fermentation, and the fatty acid compositions were considerably different for these three fermentation systems [89].

Using livestock wastewater for microalgal cultivation seems to be another alternative solution. *C. vulgaris* MBFJNU-1 in natural swine wastewater (RSW) with 3% CO_2_ resulted in the highest microalgal biomass (478.5 mg/l/day) and lipid (9.1 mg/l/day) productivities [90]. The livestock waste compost medium with 2,000 mg/l COD provided an optimal nutrient concentration for *Chlorella* sp. cultivation, where the highest productivities of biomass (288.84 mg/l/day) and lipid (104.89 mg/l/day) were achieved [91].

It is worth mentioning that free ammonia in wastewater has been demonstrated as the primary stress factor suppressing microalgal activities. Gao *et al*. reported that aeration pretreatment of SPW reduced the amount of toxic unionized ammonia to solve this problem. At the same time, most of the nutrients were retained in the wastewater [86]. An aerated biological filter (BAF), which is standard technology for aerobic biological treatment of wastewater, contains a granular media that is a collection of closely packed solid particles surrounded by a liquid media and can provide large surface areas for biofilm development [92]. BAFs have been successfully used in the traditional nitrification and denitrification processes [93]. Qin *et al*. investigated the effect of pretreatment with a BAF on microalgae culture with dairy liquid digestate. They found that the BAFs can rapidly nitrify ammonia nitrogen, eliminating ammonia inhibition for *C. pyrenoidosa* [94].

The contaminated biomass generated during phytoremediation poses a threat to our environment. Therefore, proper management is essential to dispose of the wastes to prevent them from further entering the food chain. The use and safe disposal of algal biomass after phytoremediation has been addressed by some researchers. For instance, the integration of algal bio-fertilizer production is recently gathering attention only when we use wastewater with a high level of safety to obtain pollutant-free biomass, such as wastewater from food or feed industries [95]. On the other hand, polluted water limits the application of the algae biomass produced [96]. In general, the microalgae biomass from contaminated waters can be used to produce alternative energies, including biodiesel, bioethanol, and biomethane. Additional experimentation and validation are required before the exploitation of such biomass for industrial or domestic use.

## Co-Cultivation with Bacteria, Yeast, or Other Microalgae

Co-cultivation of algae and bacteria can enhance the efficiency of nutrient utilization in wastewater, and the growth rate of microalgal cells can be improved [97]. Since various microorganisms are present in wastewater, investigating the symbiotic systems existing between microalgae and bacterial communities is necessary for developing wastewater treatment technologies. Shen *et al*. proposed a symbiotic microalgal-bacterial system using *C. vulgaris* and *Pseudomonas putida*. They revealed that the cell density of the microalgal-bacterial system was considerably increased compared to that of monoculture microalgae [98]. Moreover, Liu *et al*. reported that *Chlorella* sp. HL and three indigenous bacteria (*Brevundimonas*, *Chryseobacterium*, and *Pseudomonas*) had synergistic effects on nutrient removal [99].

Generally, the biomass produced through microalgae cultivation is harvested using processes such as centrifugation and filtration [8]. To reduce harvesting costs and remove nitrogen and phosphorous from wastewater, the floc formed by bacteria can be applied to microalgal biomass harvesting [100]. In the study by Kim *et al*., three bacterial strains (*Melaminivora jejuensis*, *Comamonas flocculans*, and *Escherichia coli*) were inoculated into a medium to form a floc with the microalgal strain *C. sorokiniana*. Among the bacterial strains tested, *M. jejuensis* formed the largest floc with *C. sorokiniana*, with the highest sedimentation ability. Furthermore, the *M. jejuensis* co-culture improved biomass and lipid productivity compared with the pure algal culture [101].

Disaccharides or polysaccharides, such as sucrose and lactose, are difficult to utilize for microalgae under heterotrophic conditions [35, 102]. Some yeasts extracellularly hydrolyze sucrose into monosaccharides. Since the sucrose hydrolysis rate is much higher than the monosaccharide uptake rate, the monosaccharide is accumulated in the culture [103, 104]. Wang *et al*. developed a co-culture system that incubates *Chlorella* with yeast *Rhodotorula glutinis* placed on immobilized beads to enhance algal growth using sucrose [105]. Tian *et al*. found that a co-culture system can enhance *Chlorella* growth using sucrose at both heterotrophic and mixotrophic modes when mix-cultured with yeast *Cryptococcus* sp. [106].

Hu *et al*. revealed that the co-cultivation system among different species of algae improved their growth. When the growth of *C. vulgaris* and a unicellular green algae *Scenedesmus dimorphus* in the landfill leachate was compared, the co-culture biomass in 10% landfill leachate demonstrated improved nutrient utilization efficiency in microalgae [107].

## Using Nanoparticles for *Chlorella* Culture 

Nanotechnology is currently a hot topic for its various applications and prospects for providing solutions to the various needs of many industries [108]. Nanoparticle application in microalgae for enhanced lipid production is an ongoing task that contributes to biodiesel production (reviewed in [109]). For example, magnesium, zinc, or lead nanoparticles induced a higher lipid content than non-metal exposed medium in *C. vulgaris*, accounting for 3.93-fold, 3.33-fold, or 2.07-fold increases, respectively [110]. Vashist *et al*. attempted to improve lipid production using silica-coated magnetic nanoparticles. As a result, silica-coated magnetic nanoparticles induced 4-fold lipid production (98 mg/l) compared to the control (28 mg/l) [111]. Moreover, MgSO_4_ was evaluated as a magnesium source for lipid production by *C. vulgaris*. The application of MgSO_4_ nanoparticles was found to improve lipid production [112]. Thus, metal nanoparticle exposure in *Chlorella* might impact various physiological or molecular changes, thereby increasing the growth rate, biomass, and lipid production.

## Various Stress Factors for Lipid Accumulation in *Chlorella*

**Fig. 3:**
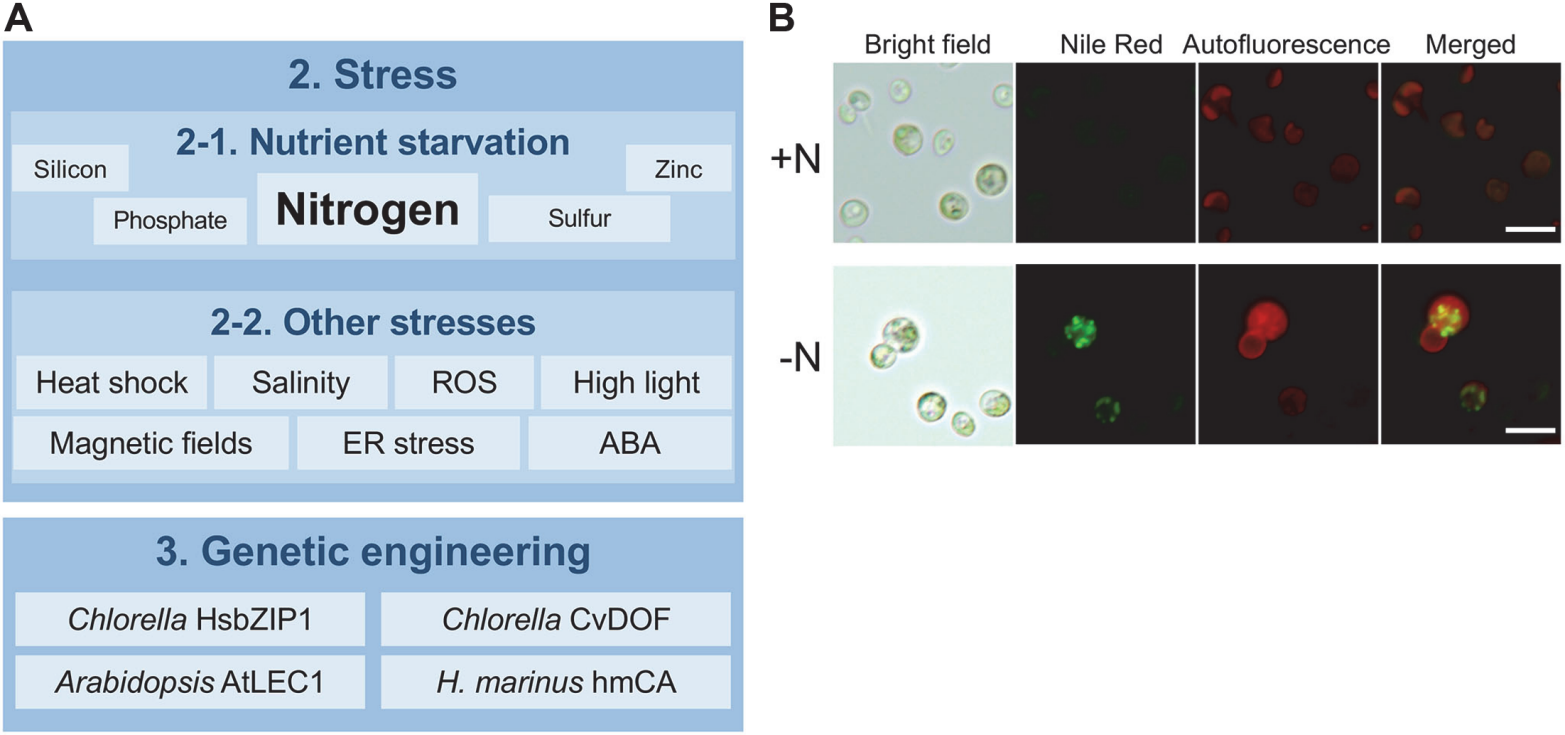
Stress factors that induce lipid production in *Chlorella* strains. (**A**) Diagram of the stress factors that induce lipid production in *Chlorella* strains. (**B**) Microscopy images of the Nile Red-stained cells grown in TAP medium with nitrogen (+N) or without nitrogen (-N) for 72 h. Brightfield, Nile Red (lipid droplets), autofluorescence, and merged images with Nile Red and chlorophyll autofluorescence are shown. Scale bars, 10 μm.

Under favorable growth conditions, *Chlorella* produces large amounts of biomass with essential lipid contents. Further induction of lipid biosynthesis by environmental stresses is a crucial step for lipid production using *Chlorella*. There has been a wide range of studies to identify and develop efficient lipid induction techniques in microalgae, such as nutrient stress (*e.g.*, nitrogen, phosphorus, sulfur starvation), osmotic stress, light, pH, temperature, heavy metals, and other chemicals (Fig. 3A).

## Nutrient Starvation for Lipid Accumulation

Nitrogen is one of the essential nutrients for the growth of microalgae. Nitrogen deprivation in microalgae is widely studied during cultivation to induce lipid productions (Table 1 in [113]). Several studies have employed a frequently used approach for increased lipid production consisting of a combination of the biomass (favorable medium) and lipid induction phase (limited nutrient medium). A commonly used two-stage strategy has been adopted for lipid induction, in which the algal cultures are harvested by centrifugation after the biomass production phase, followed by incubation in a fresh nutrient-deficient medium for the lipid induction phase [70, 114]. Due to a time- and cost-consuming harvesting process before the lipid induction stage, recently, a single-stage strategy has been getting attention, wherein nitrogen concentration in the media is adjusted to improve the overall lipid productivity. Farooq *et al*. investigated the effect of four initial nitrogen concentrations (1-, 2-, 6-, and 10-mM nitrate) on lipids yield, CO_2_ fixation rate, and water quality for further reuse after first cultivation. They concluded that the initial 6 mM nitrate was found optimum for the growth and overall lipid productivity of *C. vulgaris* [115]. Cho *et al*. observed that the effects of the initial concentrations of nitrate in the medium varied between 2.5 to 15 mM on biomass generation and lipid production of *Chlorella* sp. ABC-001, a newly isolated strain with advantageous characteristics for CO_2_ fixation and biofuel production [116]. The lipid content showed the highest value of 47.4% (DCW) with 2.5 mM nitrate, whereas the highest biomass productivity of 0.422 g/l/day was achieved under a nitrogen-rich condition (15 mM nitrate) [116].

In addition to nitrogen starvation, various nutrient starvation methods also achieved lipid stimulation in *Chlorella* [19, 117-119]. Recently, Sakarika and Kornoros optimized culture conditions for lipid accumulation under sulfur limitation, resulting in maximum total lipid content of 53.43% (DCW) [120]. Compared to nitrogen starvation, a few reports have explored the lipid contents with other nutrient-limited cultivation methods, suggesting that nutrient starvation stresses might be a powerful strategy to boost lipid production in *Chlorella* (Fig. 3B).

## Various Stresses for Lipid Accumulation

Dong *et al*. compared the effects of various stress factors on the growth and lipid production of *C. pyrenoidosa*. Their results show that the growth of *C. pyrenoidosa* was inhibited under stress conditions, but the intracellular lipid content was significantly increased. After 120 h, the greatest lipid content was under the condition of nitrogen deficiency (47.10% DCW) compared to the conditions of phosphorus deficiency (36.53% DCW), high light (34.44% DCW), high salt (28.75% DCW), and control (25.14% DCW) [121]. Gour *et al*. selected the best salinity conditions for better growth, biomass accumulation, and lipid productivity of microalgae. *Chlorella* sp. showed the maximal lipid content of 32.19% DCW and lipid productivity of 10.27 mg/l/day at 160 mM NaCl in BG-11 media. The results indicate the feasibility of enhancing the lipid content and productivity through the salinity-induced stepwise cultivation strategy [122]. Kim *et al*. treated *Chlorella* sp. HS2 with heat shock (50ºC) for 12 h and found that the algal biomass and lipid productivity were enhanced up to 4% and 17%, respectively (7.3 and 2.64 g/l/day, respectively). Thus, heat shock was successfully adopted in the novel *Chlorella* sp. HS2 cultivation for lipid induction [123]. Treatment with Brefeldin A (BFA), a chemical inducer of ER stress, triggers lipid droplet formation within 4 h in two varieties of *C. vulgaris* [124]. Zhang *et al*. treated *C. pyrenoidosa* with ferrous ions to induce reactive oxygen species (ROS) via the catalytic decomposition of hydrogen peroxide (Fenton reaction), increasing the total lipid content [125]. Abscisic acid (ABA) treatment enhanced the lipid accumulation in *Chlorella* sp. FACHB-8 strain, changing fatty acids afflux from polyunsaturated fatty acids to monounsaturated and saturated fatty acids, which were suitable for diesel application [126]. Pyrene (polycyclic aromatic hydrocarbon) is an anthropogenic organic pollutant in various ecological units. Jaiswal *et al*. used pyrene pollutants (50-500 ppm) to evaluate the impact on metabolites and the induction of lipid biosynthesis to produce renewable biodiesel. They concluded that pyrene concentration at 230 ppm caused 1.24-fold higher lipid biosynthesis compared to the control medium [127].

Research in magnetic fields has significantly affected the growth and production of proteins, carbohydrates, and lipids in microalgae. The magnetic field has been shown to have significant effects on the growth and production of proteins, carbohydrates, and lipids with *C. kessleri* [128], *Chlorella fusca* [129], and *C. vulgaris* [130]. Costa *et al*. investigated the influence of different intensities and exposure times of magnetic fields on the stimulation of lipid synthesis by the microalga *C. homosphaera*. Lipid productivity reached 39.5 mg/l/day (1.52-fold) with the magnetic fields (30 mT, 1 h/day) with a slight reduction in biomass productivity [131]. Baldev *et al*. optimized conditions of a magnetic flux density and yielded a maximum dry cell weight of 0.61 g/l, two-fold higher than the normal condition, with lipid content of 55.2% DCW, suggesting the enhancement of growth and lipid of *C. vulgaris* by magnetic fields [132].

## Genetic Engineering of *Chlorella* for Better Lipid Production

As discussed in Yang *et al*. [133], *Chlorella* transformation methods are currently considered the most severe limitation in *Chlorella* genetic engineering. Here, we introduce the latest successful examples of genetic engineering of *Chlorella* for better lipid production (Fig. 3A). Transcription factor engineering to regulate multiple genes has shown promise in the field of microalgae genetic engineering. Overexpressing *HSbZIP1*, encoding a C-type bZIP transcription factor, in *Chlorella* sp. HS2 increased fatty acid production (up to 2.13-fold) compared to control [134]. *LEAFY COTYLEDON1* (*LEC1*) is a central regulator that controls many aspects of seed development, including the maturation phase during which seeds accumulate storage macromolecules and embryos acquire the ability to withstand desiccation in *Arabidopsis thaliana* [135]. Overexpression of Arabidopsis *LEC1* in *C. ellipsoidea* enhanced the total fatty acid content (1.33-fold) and total lipid content (1.30-fold) with upregulation of key genes in the lipid synthesis pathway, such as *ACCase*, *GPDH*, *PDAT1*, and *DGAT1* [136]. In the study of Tokunaga *et al*., The candidate DOF transcription factor was endogenously overexpressed in *C. vulgaris* to improve neutral lipid production, resulting in the production of 1.5-fold higher neutral lipid content compared to control cells in *C. vulgaris* [137]. These studies provide increasing lipid content by introducing exogenous or endogenous transcription factors in *Chlorella*. The carbonic anhydrases (CA) can catalyze the rapid conversion of carbon dioxide to bicarbonate and play a key role in CO_2_ transfer for cell respiration and photosynthesis [138]. To increase the solubility of CO_2_, You *et al*. introduced carbonic anhydrase fused with dockerin to immobilize protein on the surface of *C. vulgaris*. As a result, *C. vulgaris* showed 1.6-fold rapid growth and 1.7-fold lipid production, suggesting that the CA complex can enhance CO_2_ fixation [139]. Although the success rate of heterologous gene expression remains relatively low, *Chlorella* strains harbor significant advantages for biomass and lipid production. Further improvement of *Chlorella* transformation techniques remains to be developed to provide *Chlorella* biomass as feedstock for oils, antioxidants, or other bioactive benefits by genetic engineering.

## Potential Applications of *Chlorella*

According to a market report of the Research and Market, the *Chlorella* market was worth $269.6 million in 2021, and it is expected to grow at a CAGR of 6.3% from 2021 to 2028 to reach $412.3 million by 2028 [140]. In addition, Future Market Insights reported that the global *Chlorella* market is expected to reach a market value of$198.5 million in 2022 and ultimately $427.7 million by registering a CAGR of 8% in the forecast period 2022-2032 [141]. According to another report, the global *Chlorella* market was estimated at $263.49 million in 2021. It is projected to reach $431.91 million by 2028, exhibiting a CAGR of 7.32% during the forecast period [142]. This acceleration of the *Chlorella* market growth over the forecasted period might be due to the rapidly increasing numbers of the vegan population and health-conscious consumers. According to the market report above, a high number of key participants now compete in the *Chlorella* industry. Currently, global players such as Sun *Chlorella* Corp. (Japan, https://www.sunchlorella.com), Vedan Enterprise (Taiwan, https://000527.vedan.com), FEMICO (Taiwan, http://www.femico.com.tw), and Taiwan Chlorella Manufacturing Co. (Taiwan, https://www.taiwanchlorella.com) together account for a considerably large market, where *Chlorella* is mainly sold as a dried powder, capsules, or pressed pills [58, 143]. Nutraceuticals and dietary supplements are expected to command the largest share and fastest growth in the *Chlorella* market [143].

## Valuable Compounds from *Chlorella*

Omega-3 and omega-6 fatty acids are essential to human health, and we must consume them through food; therefore, they are called essential fatty acids. Omega-3 polyunsaturated fatty acids (PUFAs) include a-linolenic acid (18:3; ALA), eicosapentaenoic acid (20:5; EPA), and docosahexaenoic acid (22:6; DHA), which are efficient at preventing cardiovascular diseases in humans, due to their characteristics that alter membrane fluidity and decrease triacylglycerols (TAGs) [144, 145]. In *C. vulgaris*, omega-3 fatty acids, such as α-linolenic acid (18:3), are dominant at around 28%, and omega-6 fatty acids account for 10% of total fatty acids [146]. Toumi *et al*. revealed that *C. sorokiniana* accumulated EPA and DHA at 16.5 and 35%, respectively, with the addition of urea [147]. Thus, *Chlorella* is an important source of essential fatty acids.

Flavonoids, secondary metabolites of plants, are involved in defense against pathogens, photosynthesis, and growth [148]. Also, flavonoids are in many human foods because of their antioxidant capacity, which can prevent ROS formation in cells [149]. Yadavalli *et al*. revealed that *C. vulgaris* and *C. pyrenoidosa* contain 138 mg/ml and 118 mg/ml of flavonoids, respectively, and these *Chlorella* species contain quercetin, catechin, and p-coumaric acid [150].

The demand for natural colorants has significantly increased due to health and environmental issues [151]. Because of the fast growth rates and diversity of pigments, microalgae have attracted great interest as a natural colorant. *Chlorella* species contain various types of pigment, including astaxanthin (red), β-carotene and violaxanthin (orange), lutein (yellow), chlorophyll-a and chlorophyll-b (green), and pheophytin-a (green-gray)[152, 153]. The amount of pigment in *Chlorella* can change by various factors, such as photoperiod, light intensity, carbon source, nitrogen source, and nanoparticles [64, 153-156].

Carotenoids have two important functions in human health as antioxidants and a precursor of vitamin A. However, humans cannot synthesize carotenoids in the de novo pathway, so it is important to consume foods containing carotenoids [158]. Carotenoids are divided into two classes, carotenes and xanthophylls [157]. Many *Chlorella* species contain xanthophylls. *C. protothecoides*, *C. sorokiniana*, and *C. vulgaris* contain lutein [159], *C. zofingiensis* contains astaxanthin [160], and *C. luteoviridis* contains zeaxanthin [161]. Also, *C. vulgaris*, *C. sorokiniana*, and *C. ellipsoidea* contain β-carotene, the precursor of vitamin A [162-164].

*C. vulgaris* contains eleven essential vitamins that humans cannot synthesize, including vitamin A, vitamin B1 (thiamine), vitamin B2 (riboflavin), vitamin B3 (niacin), vitamin B5 (pantothenic acid), vitamin B6 (pyridoxine), vitamin B9 (folate), vitamin B12 (cobalamin), vitamin C, vitamin E, and vitamin K [165-168]. Also, minerals promote several biological functions in the human body. Calcium, iron, magnesium, sodium, potassium, zinc, copper, and manganese can be found in *C. vulgaris* [166, 167]. Thus, *Chlorella* supplementation can provide various vitamin and mineral sources.

## Clinical Trial of *Chlorella* Nutritional Supplements

Based on the valuable compounds of *Chlorella*, there are already many clinical trials on how *Chlorella* affects human health. *Chlorella* supplementation in humans has been shown to have antioxidant [169], antidiabetic [170], immunomodulatory [171], and antihypertensive properties [172]. *Chlorella* intake resulted in noticeable reductions in body fat percentage, total blood serum cholesterol, and fasting blood glucose levels [173]. *Chlorella*-derived multicomponent supplementation decreases arterial stiffness in young people [174] and middle-aged and senior adults [175]. The consumption of *Chlorella* increased the level of several dicarboxylic acids in feces and propionate concentrations for individuals with low concentrations of fecal propionate [176]. These studies suggest that *Chlorella*-derived compounds might provide substitutes for synthetic compounds or drugs.

## Bioplastic Production

Bioplastics are being actively studied to eliminate the dependency on fossil fuels to produce plastics and avoid endocrine disruptors [177], and some *Chlorella* species were used in this field. *C. pyrenoidosa* can accumulate 27%poly hydroxybutyrate (PHB), a type of poly hydroxy alkanoate that is categorized as biodegradable bioplastic [178]. *C. fusca* accumulates 17.4% PHB with the addition of xylose [179].

## Taste Aspect of *Chlorella* for Plant-Based Alternatives

*Chlorella* is one of the most nutrient-dense superfoods on the earth. *Chlorella* products contain high-quality and high-quantity proteins (*C. pyrenoidosa*, 57%; *C. vulgaris*, 51%-58%) [180, 181]. Although *Chlorella* is rich in proteins, vitamins, minerals, and dietary fiber, it has been reported to have a rather bland flavor profile dominated by “grassy, vegetable, cucumber” aromas [182]. Thus, most consumers add *Chlorella* powder as an ingredient or take *Chlorella* tablets. Recently, Coleman *et al*. analyzed the taste of eight phototrophic microalgae, including *C. vulgaris*, to be used as flavor ingredients in plant-based seafood alternatives. According to their odor evaluation of eight microalgae, *C. vulgaris* has the highest earthy odor (beetroot, stale, musty odor) among the algal species they analyzed [183]. Their analysis of the chemical aroma profiles revealed that *C. vulgaris* contains higher odor activity values of alkyl aldehydes (malty/nutty/coffee) and benzaldehydes (nutty/almond) compared to the other microalgae and seafood. Although *C. vulgaris* seems to have a relatively low seafood aroma due to a lack of dimethyl sulfide, different taste evaluations disclosed that *C. vulgaris* has an intermediate level of umami (8 g MSG/100 g DW) among the algae examined with less bitterness [183]. Thus, bioengineering strategies to produce *Chlorella* strains or develop culture conditions to reduce their off-taste (earthy odor) seems necessary to use *Chlorella* as a flavor ingredient or plant-based seafood alternative.

Indeed, several companies have recently developed *Chlorella* strains to improve its taste, such as white and honey *Chlorella* (Allmicroalgae, Portugal, https://www.allmicroalgae.com), Duplaco Gold (Duplaco, Netherlands, https://duplaco.com), white *Chlorella* (Aliga Microalgae, Danmark, https://www.aliga.dk), and *Chlorella* Colors (Algenuity, England, https://www.algenuity.com). Thus, *Chlorella* seems to be a promising candidate for meeting the food needs of the vegan diet and the world’s rapidly growing population.

## Future Perspectives and Conclusion

*Chlorella*, a photosynthetic unicellular microorganism, can accelerate lipid accumulation under various environmental conditions, and has also received significant attention for biofuel production. This review has shown how *Chlorella* cultivation environments involve *Chlorella* biomass and lipid productivity, such as cultivation modes, carbon or nitrogen sources, and stress conditions. Heterotrophic cultivation with 10-20 g/l glucose seems to be used for industrial biomass production with many *Chlorella* strains. Additional glycerol will accelerate lipid production in *Chlorella* cells. *Chlorella* cultivation with a light source will induce more antioxidant content. Although *Chlorella* will assimilate nitrogen from either nitrate or ammonium, ammonium can be assimilated with less energy in *Chlorella* cells. Moreover, it is worth mentioning that we must carefully select nitrogen sources based on the genetic information of each *Chlorella* strain. Nitrogen starvation is an efficient environmental pressure for boosting lipid accumulation in *Chlorella* cells.

The current severe bottleneck includes the high manufacturing cost of *Chlorella* biomass production, harvesting, and processing. To solve these issues, we need to develop a cost-effective method of culturing *Chlorella* species, such as *Chlorella* with high biomass productivity, high photosynthetic efficiency with high density and high tolerance to various harsh environments (pH, temperature, or osmolarity). Also, we need to consider how to increase the production of value-added ingredients that can cover high manufacturing costs. Development of *Chlorella* genomic databases might also support genetic engineering approaches for those strain developments. Although *Chlorella* has come under the spotlight for its potential as a sustainable, nutrient-rich future food solution, it has not yet won over the consumer’s taste buds due to its earthy and grassy smell and taste. It is essential to provide *Chlorella* strains without an unpleasant taste for use in food.

Overall, *Chlorella* is a valuable alga that has two attractive consumption purposes as a potential source of renewable energy and nutrient-rich food. The knowledge and techniques accumulated in both fields will be utilized to develop innovative applications and culturing methods.

## Figures and Tables

**Table 1:** *Chlorella* biomass and lipid productivity using different carbon sources.

Mode	Carbon source	Carbon	Strain	Medium	Culture volume	Biomass	Unit	Lipid	Unit	Ref.	
Autotrophic	CO2	Nitrogen depletion	*C. minutissima*	Guillard f/2		62.5	mg/L/d	5.72	mg/L/d	[28]	Amaral *et al*., 2020
Autotrophic	CO2	Nitrogen depletion	*C. pyrenoidosa* 2738	Fog’s media		5	g/L/13d	410	mg/L/d	[16]	Nawkarkar *et al*., 2019
Autotrophic	CO2	-	*C. sorokiniana* AM-02	BBM	2.4 L	3.45	g/L	NA		[30]	Ziganshina *et al*., 2020
Heterotrophic	Glucose	10 g/L glucose	*C. vulgaris* AB MCCS 130	BG11	2 L	265	mg/L/d	118	mg/L/d	[32]	Morowvat *et al*., 2019
Heterotrophic	Glucose	10 g/L glucose	*Chlorella* sp. HS2	BG11	3 L	5370	mg/L/d	860	mg/L/d	[33]	Kim *et al*., 2019
Mixotrophic	Glucose	15 g/L glucose	*C. vulgaris* KNUA104	BG11		2.98	mg/L/d	68.80%	DCW	[38]	Yun *et al*., 2021
			*C. sorokiniana* KNUA122			4.73		40%*			
Heterotrophic			*C. vulgaris* KNUA104			1.72		30%*			
			*C. sorokiniana* KNUA122			3.64		40%*			
Mixotrophic	Glucose	18.8 g/L glucose	*C. vulgaris* strain UTEX 2714	TAP	150 mL	6.1	g/L	383	mg/L/d	[39]	Ward *et al*., 2019
Mixotrophic	Acetate	10 g/L acetic acid	*C. pyrenoidosa* (FACHB-1216)	BG11	800 mL	134	mg/L/d	42.04	mg/L/d	[54]	Li *et al*., 2022
Mixotrophic	Acetate	100 mM acetic acid	*C. sorokiniana* 211-32		250 mL	1390	mg/L/d	193.37	mg/L/d	[40]	León-Vaz *et al*., 2019
Mixotrophic	Acetate	10 g/L NaAc	*C. pyrenoidosa* (FACHB-9)	BG11	300 mL	40*	mg/L/d	13.48	mg/L/d	[41]	Liu *et al*., 2018
Mixotrophic	Glycerol	3 g/L glycerol (synthetic wastewater)	*C. pyrenoidosa*	-	3.5 L	1.28	g/L	30.76%	DCW	[45]	Rana *et al*., 2021
Heterotrophic	Glucose	20 g/L glucose	*C. vulgaris* CCALA 256	BBM	2 L	NA		32.70%	DCW	[55]	Canelli *et al*., 2020
Mixotrophic								24.20%			
Mixotrophic	Wastewater	25% Sweet sorghum bagasse (SSB)	*C. vulgaris* UTEX 395	BBM	2 L	3.44	g/L	141	mg/L/d	[80]	Arora *et al*., 2021
Mixotrophic	Wastewater	Food waste extract (20 g/L glucose)	*Chlorella* sp. GY-H4	-	2 L	6.9	g/L	1.8	g/L	[86]	Wang *et al*., 2020
Mixotrophic	Wastewater	30% Palm oil mill effluent (POME)	*C. sorokiniana* CY-1	-	7.02 L	409	mg/L/d	14.43%	DCW	[81]	Cheah *et al*., 2020
Heterotrophic	Wastewater	Sugarcane bagasse (20 g/L sugar conc)	*C. protothecoides*	-	7 L	10.7	g/L	16.80%	DCW	[83]	Chen *et al*., 2019
Heterotrophic	Wastewater	Forest biomass (C/N 60)	*C. sorokiniana* SAG 211–8 k	-	1.9 L	8.28	g/L	3.61	g/L	[84]	Vyas *et al*., 2022
Autotrophic	Wastewater + CO2	Seafood processing wastewater (SPW) + 10% CO2	*C. vulgaris* NIOCCV	-	4 L	264	mg/L/d	100.54	mg/L/d	[85]	Jain *et al*., 2019
Mixotrophic	Wastewater	Seafood processing wastewater (SPW)	*Chlorella* sp.	-	350 mL	77.7	mg/L/d	20.4	mg/L/d	[86]	Gao *et al*., 2018
Mixotrophic	Wastewater	Tannery effluent : sewage effluent = 20 : 80	*C. vulgaris*	-	300 mL	3.25	g/L	25.40%	DCW	[87]	Saranya *et al*., 2019
			*C. pyrenoidosa*			2.84		9.30%			
Mixotrophic	Wastewater	OSCCW : Water = 50 : 50	*C. vulgaris* (NRMCF0128)	-	-	60.1	mg/L/d	20.8	mg/L/d	[88]	Azam *et al*., 2022
			*P. pringsheimii* (VIT_SDSS)			56.5		17.5			
Mixotrophic	Wastewater	Butyric acid type effluent	*Chlorella* sp. UJ-3	-	200 mL	NA		25.40%	DCW	[89]	Huo *et al*., 2018
Autotrophic	Wastewater + CO2	Real swine wastewater (RSW) + 3% CO2	*C. vulgaris* MBFJNU-1	-	3000 L	478.5	mg/L/d	9.1	mg/L/d	[90]	Xie *et al*., 2022
Mixotrophic	Wastewater	2000 mg/L COD	*Chlorella* sp.	-	225 mL	288.84	mg/L/d	104.89	mg/L/d	[91]	Zhu *et al*., 2017
Heterotrophic	Sucrose + yeast	10 g/L sucrose	*C. pyrenoidosa*	BG11	100 mL	2290	mg/L/10d	124.3	mg/L/d	[104]	Kilian *et al*., 1996
Mixotrophic			+ *Cryptococcus* sp.			2930		165.4			
Heterotrophic	Sucrose + yeast	1% sucrose	*C. pyrenoidosa* FACHB-9	BG11	100 mL	340	mg/L/d	29.70%	DCW	[103]	Wang *et al*., 2016
			+ *Rhodotorula glutinis*								

Detailed conditions of carbon treatments for the accumulation of lipids in *Chlorella*.

*indicates value estimated from figure images.

**Table 2:** *Chlorella* biomass and lipid productivity using different nitrogen sources.

Nitrogen Source	Nitrogen	Strain	Medium	Culture volume	Growth rate	Unint	Biomass	Unit	Lipid	Unit	Protein	Unit	Ref	
Nitrite	Nitrite 0% (Nitrite+Nitrate)	*C. vulgaris*	B3N	50 mL	1.3	day	NA		NA		NA		[64]	Pozzobon *et al*., 2021
Nitrate+Nitrite	Nitrite 20-100% (Nitrite+Nitrate)				0.82-0.97									
Nitrate	NA	*C. vulgaris*	Jaworsky	8 L	NA	day	0.18	g/L	12.29%	DCW	**50.80%**	DCW	[66]	Mutlu *et al*., 2011
Nitrate+Nitrite							0.12		13.04%		41.03%			
–Nitrogen							0.18		35.60%		13.01%%			
Nitrite	15 mg N/l	*Chlorella* sp. HQ	mBG11	180 mL	NA	day	342.5	mg/L	**38.75**	mg/L	NA		[67]	Zhan *et al*., 2016
Nitrate							357.5		19.99					
Ammonium							102.5		5.86					
Urea							270		12*					
Nitrite	200 umol/L Nitrite	*Chlorella* sp. L38	BG11	1 L	NA	day	NA		**3.05**	mg/L/d	2.67	mg/L/d	[65]	Li *et al*., 2020
Nitrate+Nitrite	200 umol/L Nitrate+Nitrite							1.15		5.06				
Nitrite	0.8 g N/L	*Chlorella* sp. GN1	BG11	1 L	0.947	day	216	mg/L/d	NA		NA		[70]	Feng *et al*., 2020
Urea					**1.9**		**345**							
Ammonium					0.726		170							
Urea	17.6 mM N	*Chlorella* sp. HS2	BG11	100 mL	**0.765**	day	**301.4**	mg/L/d	**63.6**	mg/L/d	NA		[70]	Nayak *et al*., 2019
Sodium nitrate					0.751		274.3		37.7					
Potassium nitrate					0.733		241.1		52.9					
Ammonium nitrate					0.415		24.3		4.2					
Ammonium chloride					0.387		21.4		3.4					
Ammonium acetate					0.741		255.7		50.4					
Ammonium sulfate					0.421		27.1		4.9					
Ammonium bicarbonate					0.696		187.1		37.9					
Ammonium	~215 mg/L Ammonium	*C. sorokiniana* AM-02	BBM	2.6 L	**1.26**	day	NA		NA		NA		[74]	Wang *et al*., 2019
Nitrate	~730 mg/L Nitrate				1.07									

Detailed conditions of nitrogen treatments for biomass, lipid and protein productions in *Chlorella*.

* indicates value estimated from figure images. Numbers discussed in the text are in bold.
